# Potential factors that influence usage of complementary and alternative medicine worldwide: a systematic review

**DOI:** 10.1186/s12906-020-03157-2

**Published:** 2020-11-23

**Authors:** Mayuree Tangkiatkumjai, Helen Boardman, Dawn-Marie Walker

**Affiliations:** 1 Department of Clinical Pharmacy, Faculty of Pharmacy, Srinakharinwirot University, Nakhonnayok, 26120 Thailand; 2 Division of Pharmacy Practice and Policy, School of Pharmacy, University of Nottingham, Nottingham, UK; 3 School of Health Sciences, University of Southampton, Southampton, UK

**Keywords:** Conventional medicine, Complementary and alternative medicine, Use, Not use, Factor

## Abstract

**Objectives:**

To determine similarities and differences in the reasons for using or not using complementary and alternative medicine (CAM) amongst general and condition-specific populations, and amongst populations in each region of the globe.

**Methods:**

A literature search was performed on Pubmed, ScienceDirect and EMBASE. Keywords: ‘herbal medicine’ OR ‘herbal and dietary supplement’ OR ‘complementary and alternative medicine’ AND ‘reason’ OR ‘attitude’. Quantitative or qualitative original articles in English, published between 2003 and 2018 were reviewed. Conference proceedings, pilot studies, protocols, letters, and reviews were excluded. Papers were appraised using valid tools and a ‘risk of bias’ assessment was also performed. Thematic analysis was conducted. Reasons were coded in each paper, then codes were grouped into categories. If several categories reported similar reasons, these were combined into a theme. Themes were then analysed using χ^2^ tests to identify the main factors related to reasons for CAM usage.

**Results:**

231 publications were included. Reasons for CAM use amongst general and condition-specific populations were similar. The top three reasons for CAM use were: (1) having an expectation of benefits of CAM (84% of publications), (2) dissatisfaction with conventional medicine (37%) and (3) the perceived safety of CAM (37%). Internal health locus of control as an influencing factor was more likely to be reported in Western populations, whereas the social networks was a common factor amongst Asian populations (*p* < 0.05). Affordability, easy access to CAM and tradition were significant factors amongst African populations (*p* < 0.05). Negative attitudes towards CAM and satisfaction with conventional medicine (CM) were the main reasons for non-use (*p* < 0.05).

**Conclusions:**

Dissatisfaction with CM and positive attitudes toward CAM, motivate people to use CAM. In contrast, satisfaction with CM and negative attitudes towards CAM are the main reasons for non-use.

**Supplementary Information:**

The online version contains supplementary material available at 10.1186/s12906-020-03157-2.

## Background

Use of complementary and alternative medicine (CAM) has become widespread in the last two decades. The prevalence of CAM use in general populations worldwide ranges from 9.8% to 76% [1]. Twelve systematic reviews report reasons for CAM use mainly in cancer populations compared to other condition-specific populations [2–5].

Five of the systematic reviews aimed to determine reasons for CAM use in either general or condition-specific populations [2, 5–8]. The reviews reported that the main reasons for CAM use were: (a) expected benefits and perceived safety of CAM, (b) control and participation in their therapy, and (c) alignment of socioculture, beliefs and needs. The other six reviews also reported reasons for CAM use, but this issue was not their main aim [3, 4, 9–12]. Their findings showed various reasons for CAM use, such as: (1) the benefits and safety of CAM, (2) availability and accessibility of CAM, (3) influence from friends, family, and the mass media, and (4) dissatisfaction with conventional medicine (CM). One systematic review from sub-Saharan Africa also reported barriers to CAM use that included: (a) the absence of conclusive scientific evidence for CAM, (b) a lack of belief in safety and efficacy of CAM, and (c) unhygienic practice in product preparation [9].

A narrative review (Jones et al., 2019) aimed to determine factors influencing CAM use in Australia and reported that cancer and other condition-specific populations shared some reasons for CAM use: (a) self-perceived ill health, (b) sense of well-being and (c) integrative treatment [13].

However these reviews do not directly compare similarities and differences in the reasons for CAM use between populations. There are also limited systematic reviews reporting reasons for not using CAM. The present review aimed to provide comprehensive understanding of factors influencing different populations to use/not use CAM.

## Methods

The Preferred Reporting Items for Systematic Reviews and Meta-Analyses (PRISMA) 2009 statement was employed in the present systematic review [14]. Research questions of this review were 1) What were the similarities and differences in reasons for using/not using CAM amongst general and condition specific populations? and 2) What were the similarities and differences in reasons for using/not using CAM amongst populations in each region?

### Search strategy

The databases – PubMed: National Library of Medicine, ScienceDirect and EMBASE were searched. It is recommended that two or more databases are searched. EMBASE alone has the highest percentage recall of papers and, as a result, gains in searching resources beyond EMBASE are modest [15–17]. Keywords used were ‘herbal medicine’ OR, ‘herbal and dietary supplement’ OR, ‘complementary and alternative medicine’, AND ‘reason’ OR ‘attitude’. Free-text terms combined with Boolean operators and filters were used for searching relevant studies [18]. For example, ‘complementary and alternative medicine’ AND ‘reason’; ‘complementary and alternative medicine’ AND ‘attitude’. All permutations of these key words were performed. Herbal medicine and dietary supplements were used as keywords due to these products being extensively used worldwide, compared to other types of CAM [19–25]. Pubmed and EMBASE were chose because they are the main sources suggested by the Cochrane centre and provide relevant studies in this field [26]. Meanwhile, the ScienceDirect database has published information relating to the social sciences A date range of January 2003 to December 2018 was set as the World Health Organization’s (WHO) 2002 definition of CAM was used to underpin this research: “CAM are used to refer to a broad set of health care practices that are not part of a country’s own tradition, or not integrated into its dominant health care system” [27]. This current review began in 2019 and has reviewed relevant sources published ovevr a 15 year period from 2003 to 2018.

### Selection criteria

Original articles published in English from 2003 to 2018 were reviewed. Quantitative, and qualitative studies, and mixed-methods research were included as each type of publication provided a different informational perspective and complemented each other. No limits were set regarding country of origin or type of population. This process was conducted by two independent reviewers.

### Exclusion criteria

Conference proceedings, pilot studies, study protocols, letters, literature reviews or systematic reviews were excluded. The studies which did not report on factors or reasons for using, or not using, CAM were excluded. Furthermore, papers which studied some specific groups were also excluded, i.e. students, medical professionals, pregnant women, people aged less than 15 years, care givers, or specific sexual identities or ethnic groups. This exclusion was due to the premise that each group has a specific characteristic which may underpin their reasons for CAM usage, which may deviate from other populations. As the present review focused on the reasons and attitudes influencing people to use/not use CAM, efficacy trials of CAM were also excluded.

### Data extraction and risk of bias assessment

The process of extracting data from publications was conducted by two independent reviewers. Any disagreements were resolved by discussion with a third reviewer. The included quantitative studies were appraised using a standard tool adapted from Gan’s study, which contained 10 items and assessed a study’s internal and external validity [22]. Meanwhile, the qualitative studies were assessed by a standard tool from Jakes’ study, which evaluated agreement between research questions, methods, representation, interpretation of results, influence of researchers, evidence of ethical approve and a flow from the analysis to conclusion [12]. These tools have been used for evaluating studies in the CAM field and seem to be appropriate to assessing the methodologies of the observational and qualitative studies included in this present systematic review.

### Data synthesis and statistical analysis

Data in the present systematic review was analysed by both qualitative and quantitative methods conducted by two independent reviewers [28]. An inductive thematic approach was performed to identify themes of reasons for use and non-use of CAM [28]. All use or non-use reasons in each publication were coded by hand, and then grouped into a category according to the reason(s). If several categories reported similar reasons, such categories were combined into one theme. The themes, therefore, emerged from this process. This process was forward and backward analysed until the themes were consistent. Then, similarities and differences of the themes between general and condition-specific populations, and between Western and Asia populations were analysed by χ^2^-tests. Tests were two-tailed and a *p*-value < 0.05 was considered statistically significant.

## Results

**Fig. 1:**
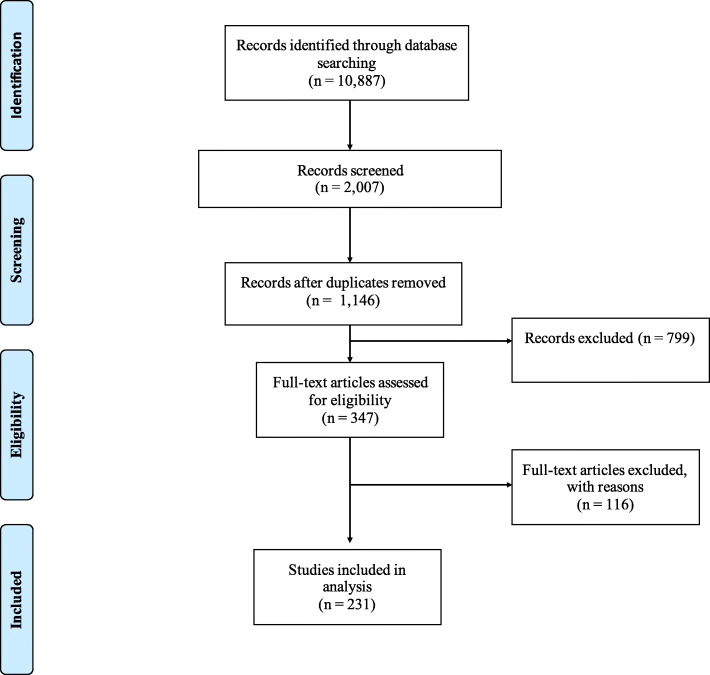
Flowchart of study identification process

Searching via the three databases provided 10,887 publications. After excluding irrelevant publications based on their title and abstract, 2,007 publications remained, from which 861 duplicates were removed. 799 publications met the exclusion criteria and were therefore not included. From the 347 full-text articles reviewed, 116 publications were excluded due to an absence of reporting factors or reasons for CAM use, resulting in 231 publications from 51 countries being included in the analysis (Fig. 1).

Thirty-seven out of the 231 included publications were qualitative studies (16%) mainly from the United Kingdom (UK) [29–34], the United States (US) [35–38], Australia [39–41] or Canada [42–44]; a survey or cross-sectional study were the most commonly employed quantitative methods in the included publications (80.5%). Only eight mixed method papers (3.5%) reported the reasons for CAM use [32, 45–51]. Eleven papers (4.8%) were conducted in elderly populations [35, 39, 42, 43, 52–58], and six (2.6%) in women only populations [29, 38, 39, 59–61].

The highest number of all included publications originated in Asia (25.5%), followed by Europe (20.9%), North America (20.0%) and the Middle East (14.7%). A small number of publications from Australia were also included in the present systematic review (7.8%).

To gather information the majority of the quantitative papers utilised questionnaires which provided a list of factors or reasons for using CAM based on previous studies. The majority of the included qualitative studies utilised interviews or focus groups with open-ended questions and employed thematic or inductive analyses. Sixty-four percent of the included publications defined CAM based on the National Center for Complementary and Alternative Medicine (NCCAM), the World Health Organization (WHO, 7%), and the others, e.g. the Food and Drug Administration, the Dietary Supplement and Health Education Act (DSHEA), Ernst’s definition, Eisenberg’s definition, etc.

**Fig. 2:**
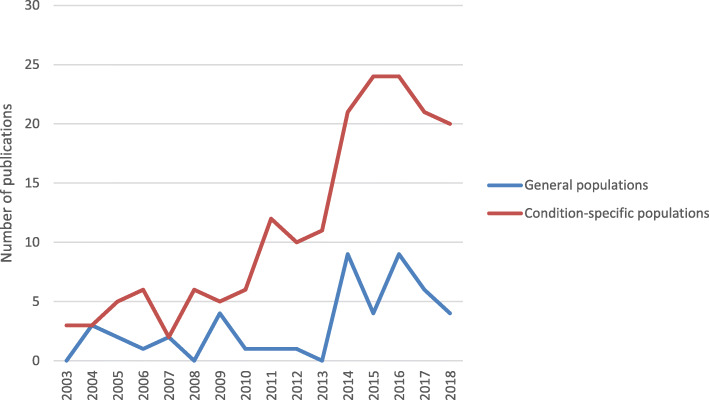
Trend in numbers of the publications of reasons for CAM use

Figure 2 shows an increase in the number of publications related to reasons for CAM use amongst condition-specific populations since 2013, compared with publications involving general populations. The total number of publications dealing with CAM use amongst general and condition-specific populations in this review was 48 (21%) and 179 (77%), respectively. The number of the papers in condition-specific populations is higher than in general populations (Fig. 2), i.e. cancer (29.0% of publications), diabetes (5.6% of publications), cardiovascular disease and hypertension (5.2% of publications), human immunodeficiency virus (HIV) (3.5% of publications), inflammatory bowel disease (3% of publications), pain (3% of publications), chronic kidney disease (2.6% of publications), and depression (0.9% of publications). The majority of studies in the present review reported various types of CAM use (69%), followed by herbal medicine (18%) and traditional medicine, including traditional Chinese medicine (1%).

The risk of bias assessment resulted in one quantitative publication being excluded due to poor internal and external validity. The included studies addressing general populations had a low bias risk (mode of a total score = 10, range 7-10), and for condition-specific populations there was a moderate risk of bias (mode of a total score = 7, range 5 – 10). The weaknesses of studies involving condition-specific populations was mainly due to a lack of reporting of their randomisation procedure (73% of the publications), how representative the sample was (64%), and non-response bias (48%). Details of the risk of bias assessment provided in a supplementary material no. 1.

Lack of reporting the researcher’s background (69% of the publications) and the influence of reseachers on the research (60%) were the main weaknesses of the included qualitative studies in both general and condition-specific populations.

### Themes of reasons for use and non-use of CAM

**Fig. 3:**
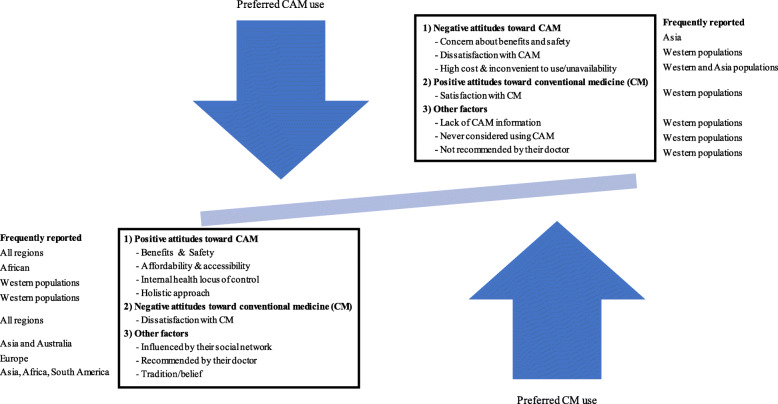
Factors related to reasons for CAM use and non-use

Both quantitative and qualitative studies reported similar reasons for CAM use. Thirty-three (14.3%) publications provided reasons for use as well as non-use of CAM. The present systematic review found three main factors related to reasons for CAM use: positive attitudes toward CAM, negative attitudes toward CM, and other factors, i.e. influence of their social network, their doctor’s recommendation, having an internal health locus of control and tradition (Fig. 3). Reasons for non-use of CAM were having negative attitudes toward CAM and positive attitudes toward CM.

### Reasons for CAM use amongst general and condition-specific populations

There was no difference in reasons for CAM use between general and condition-specific populations. The top three reported reasons for CAM use in all populations were perceived benefits (84% of publications), and safety of CAM (37%), and dissatisfaction with CM (37%). The most reported expected benefits of CAM were treatment of illnesses, alleviation of symptoms, reducing side effects of CM, maintenance of well-being, or prevention of disease. People also reported that using CAM was a last resort [29, 44, 52, 62–64]. Improving physical and emotional well-being, and quality of life were further reasons for using CAM in patients with cancer [50, 63, 65–72]. The cancer patients also reported using CAM to reduce side effects of CM [33, 45, 65, 66, 69, 73–80]. Western populations in both the general and condition-specific populations were more likely to report combining CAM and CM helped them [33, 54, 74, 81–84]. Likewise, condition-specific populations in some Asian and Middle East countries perceived that CAM complemented CM [63, 85–89]. Even though CAM is more likely to be a mainstream therapy in Asian countries, the Asian condition-specific populations tend not use CAM as a substitute for CM. However, CM is substituted with CAM amongst general populations in Japan [90].

Regarding dissatisfaction with CM, being ineffective and/or causing side effects were the most frequently reported reasons in both general and condition-specific populations for their lack of satisfaction [29, 36, 40, 54, 81, 87, 91–107]. Some patients wanted to use CAM in order to either avoid side effects resulting from CM or to decrease the number of conventional medicines taken [49, 92, 103, 108–112]. A lack of trust in CM as the reason for using CAM was reported in three publications from Asia, two from the Middle East and one from Europe [61, 94, 113–116]. Additionally, condition-specific populations decided to use CAM to avoid invasive care or aggressive treatment [80, 111]; or they were disappointed with or had negative experience of conventional care and/or the staff providing it [41, 80, 86, 97, 103, 105, 117–119]. CAM users in both Asian and Western populations preferred to visit CAM practitioners because they provided fuller explanations and more time when compared with conventional health professionals [34, 66, 86]. Condition-specific populations in Asia and Africa often found it difficult to access CM; a circumstance which drove them to use CAM [97, 120].

Only 8.7% of publications found that condition-specific populations viewed CAM as natural, and thus safe [21, 29, 49, 65, 67, 76, 92, 100, 106, 107, 112, 114, 121–129]. Six studies from Europe, Asia and Africa also reported ‘being curious’ as the reason for using CAM [92, 97, 130–133].

Other factors were influenced by CAM users’ social networks (27% of publications), having an internal health locus of control defined as preferring to control or decide choices of health treatments themselves (28%), affordability of CAM (24%), willingness to try or use CAM (including hope) (21%), conventional health professionals’ recommendation (18%), easy access to CAM (14%), belief in a holistic approach (12%), and tradition/belief (12%). Internal health locus of control and a holistic approach were more likely to reported by Western populations, as such reasons may be developed from or informed by a Western perspective [30, 31, 34, 37, 42, 45, 46, 48, 50, 55, 56, 67, 68, 71, 73, 80, 91, 93, 95, 106, 107, 125–128, 130, 134–159].

### Similarities and differences in reasons for CAM use amongst patients with cancer and other chronic illnesses

**Fig. 4:**
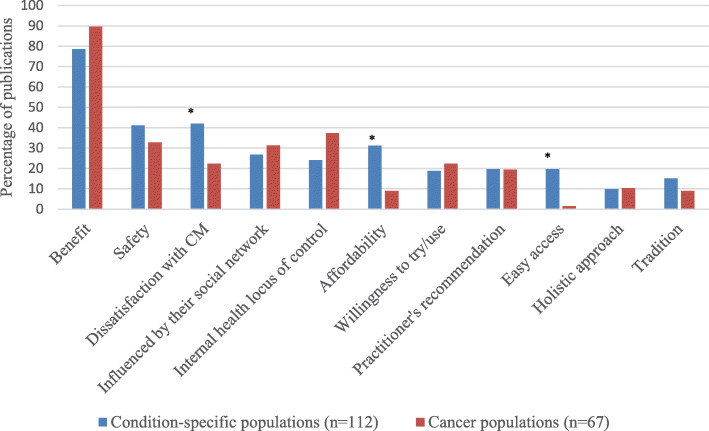
Comparing the reasons for CAM use amongst cancer patients and patients with other chronic illnesses. * Statistical significant at *p* < 0.05

The literature shows that patients with various illnesses share the main reasons for CAM use, such as perceived benefits of CAM use or dissatisfaction with CM rather than having different reasons in specific diseases [36, 40, 41, 48, 57, 62, 84, 86, 87, 92, 96, 99, 104, 108, 125, 160–167]. However, being influenced by social media, having an internal health locus of control, or willingness to try CAM were reported more frequently by cancer patients than other members of condition-specific populations (Fig. 4). Meanwhile dissatisfaction with CM, affordability of CAM and easy access to CAM were more frequently reported by patients with other chronic illnesses (*p* < 0.05). Patients with cancer, whilst accepting the efficacy and safety of CM, may use CAM in order to complement the efficacy of chemotherapy and/or reduce its unpleasant side effects (36% of publications in cancer populations) [33, 34, 45, 63–67, 69, 71, 73–79, 88, 122–124, 139, 152].

### Reasons for CAM use in each region

**Fig. 5:**
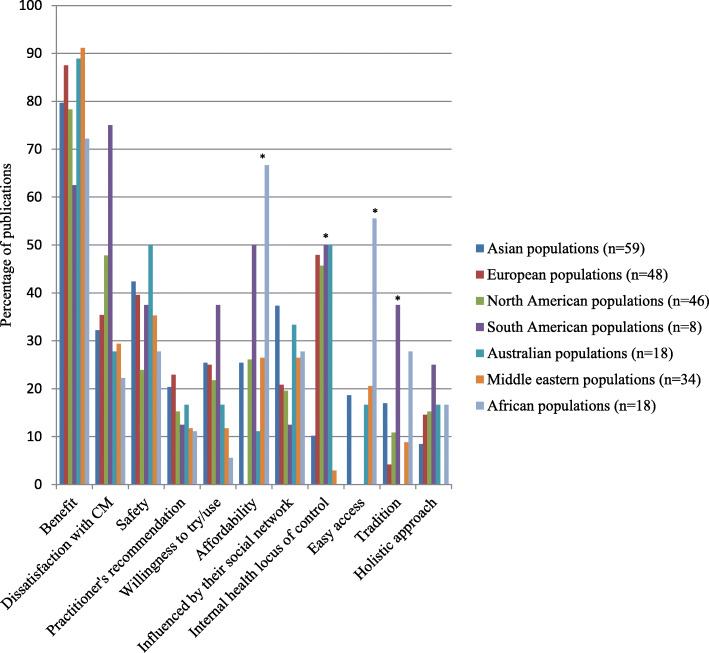
Comparison of the reasons for CAM use worldwide. * Statistical significant at *p* < 0.05

There was no global difference in the reported reasons for using CAM, namely the benefits of CAM, dissatisfaction with CM, and safety of CAM, see Fig. 5. However, the number of publications in Europe (35% of publication), North America (48%) and South America (75%) that reported dissatisfaction with CM as the reason for using CAM was higher than in other populations. The benefits (89% of publications) and safety of CAM (50%) were reported as of the main reasons for CAM use in Australian populations.

An internal health locus control, affordability and easy access of CAM, as well as tradition/belief were significantly different in each region (*p* < 0.05). Internal health locus control influenced people in Australia (50% of publications), South America (50%), and Europe (48%). Additionally, tradition significantly influenced CAM use in South America (38% of publications), Africa (28%) and Asia (17%), compared with other regions. A high proportion of publications in Asian (37%) and Australian populations (33%) reported that social networks influenced them to use CAM, compared with other regions. African populations had the highest proportion of reported affordability of CAM (67%) and easy access (56%) as reasons for CAM use, whilst no report of these reasons was found in European populations. European populations (23% of publications) are more likely to report conventional health professionals’ recommendations for CAM use as their reason, compared with other regions.

**Fig. 6:**
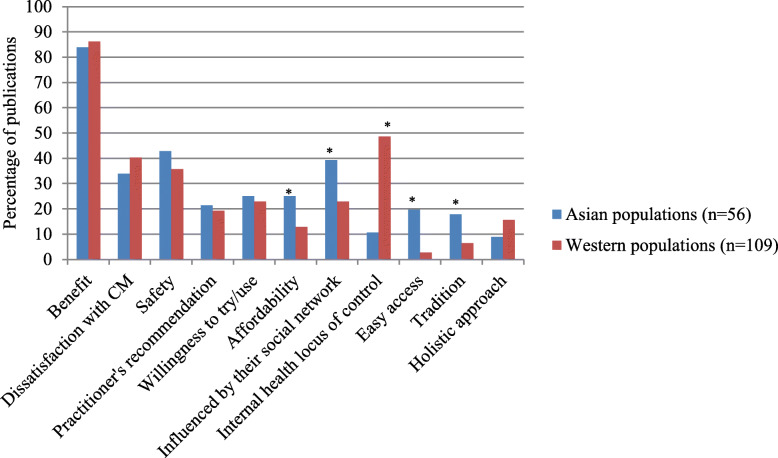
Comparison of the reasons for CAM use between Asian and Western populations. * Statistical significant at *p* < 0.05

Regarding reasons for CAM use amongst Western and Asian populations, Asian populations more frequently reported using CAM due to being influenced by members of their social network, low costs of CAM, easier access to CAM and tradition than Western populations (*p* < 0.05), Fig. 6. Meanwhile, having an internal health locus of control is the main reason for CAM use in Western populations (*p* < 0.05).

### Reasons for non-use amongst Western and Asian populations

The studies of reasons for non-use are limited compared to the reasons for using CAM, so comparison of the reasons for non-use in each region cannot be made. No publications from the Middle East or South America were included in the present systematic review. Two publications from Africa and Australia were included. The majority of studies in the included publications were conducted in Asia, Europe or North America. Therefore, we compared Asian and Western populations.

**Fig. 7:**
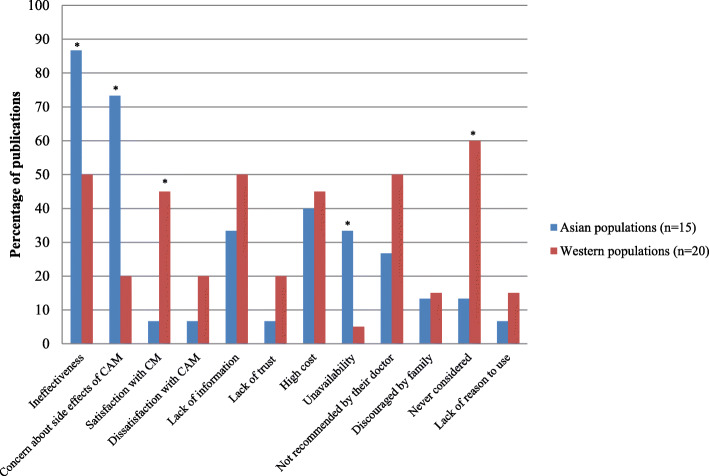
Reasons for non-use between Asian and Western populations. * Statistical significant at *p* < 0.05

Thirty papers in condition-specific populations [45, 47, 65, 75, 79, 80, 95, 107, 115, 120, 123, 124, 130, 150, 152, 157, 162, 168–179], six in general populations [180–185], one publication involving elderly people [53] and one publication involving females [61] reported the reasons for not using CAM. Asian populations more frequently reported doubt about the efficacy of CAM or lower effectiveness of CAM compared to CM, concerns about side effects of CAM, and inconvenience or unavailability of CAM than did members of Western populations (*p* < 0.05), Fig. 7 [47, 75, 79, 120, 170, 172, 174–177, 179–181]. Some publications in Asian populations also reported concern about CAM reducing the efficacy of CM as a reason for non-use [169, 170].

Meanwhile, Western populations mainly reported satisfaction with CM (45% of publications, *p* < 0.05) or had never considered using CAM (60%, *p* < 0.05) [45, 65, 95, 123, 124, 130, 150, 152, 157, 162, 171, 182, 184, 186]. Other reasons for the non-use of CAM were lack of reliable information about the efficacy of CAM, the high cost of CAM, and it not being recommended by conventional health professionals or the ‘patient’s’ family.

## Discussion

The included studies in the present systematic review can be seen to represent CAM use worldwide as they were mainly from Asia, Europe, North America, Middle East, and Australia. Recently, researchers in Asia have become interested in this field as several CAMs are embedded in their culture and society. Publications from this region has been rising since 2008; however, readers should be aware that the present systematic review included a small number of eligible publications from Australia and South America. Although a high number of publications originated in Australia, they tended to study specific populations, e.g. middle-aged women, and other topics, rather than reasons for CAM use. Researchers may less likely to investigate reasons for CAM use in South America, compared to other regions. Therefore, the findings in the present systematic review may be less likely to be generalisable in Australian and South American populations.

The present systematic review included a high number of publications amongst cancer, diabetic, cardiovascular disease, and HIV populations. It would therefore seem that illnesses, such as these which cannot be satisfactorily treated by CM, or when CM has significant unpleasant side-effects, drive some patients to seek CAM. Cancer populations have been studied regarding reasons for CAM use more than other condition-specific populations. There are six systematic reviews of the reasons for CAM use in patients with cancer [2, 3, 5, 8, 187, 188].

As expected, three main factors related to reasons for CAM use in the present systematic review were positive attitudes toward CAM, negative attitudes toward CM and other factors, i.e. the influence of their social network, their doctor’s recommendation, having an internal health locus of control and tradition. The top three reported reasons for CAM use were perceived benefits and safety of CAM, and dissatisfaction with CM. These findings are consistent with previous systematic reviews [3, 9–12]. These reasons are similar in both general and condition-specific populations, and in populations from different global regions, as cited frequently above. Although the present systematic review included a limited number of publications from Australia, benefits and safety of CAM were reported as the main reasons for CAM use in Australian populations. These findings agree with a previous systematic review in Australia [6].

Despite limited scientific evidence for the benefits of CAM [189], the ‘expected benefits’ of CAM was the most frequently reported reason for CAM use. This finding is not surprising as people tend to seek CAM as a way of meeting their needs or filling a gap left by conventional medicine. The included publications amongst the cancer population are more likely to report CAM use for reducing the negative and often unpleasant side effects of CM. This finding is consistent with systematic reviews of CAM users with prostate or advanced cancer [3, 4]. Additionally, the cancer population seems to accept the efficacy and side effects of CM, and therefore uses CAM to complement CM.

Previously, people believed that CAM is natural and safe [190]. This idea may have led many patients with chronic illnesses on using CAM instead of CM. However, the present systematic review indicates that a small number of the included publications amongst condition-specific populations reported that CAM is safe as a reason for CAM use. Therefore, CAM as natural therapy is not the main reason for CAM use; a point which may be linked to the high number of reported adverse events from using CAM [191–193]. Patients therefore should use CAM with caution or under supervision from conventional or CAM practitioners.

Regarding other factors related to CAM use in each region, nearly half of the included publications reported that internal health locus control influenced people in Australia, South America, and Europe. However, this reason may have been reported less by Asian, Middle Eastern and African people, as they may not explain their reasons in such terms. Tradition also significantly influenced CAM use in South America, Africa and Asia, compared with other global regions. This orientation may be because CAM, for example, herbal medicine, is embedded in such regions and therefore aligns with their populations’ socio-culture values. Social networks influenced Asian and Australian populations to use CAM, compared with other regions, as they may have a close-knot family or community structure.

Affordability of CAM, together with easy access, are likely to be the main reasons for CAM use amongst African populations. The high cost of, and poor accessibility to, CM appears to influence people to use CAM in Africa [57, 58, 97, 164, 183, 194–201]. Meanwhile, no report of these reasons was found in European populations. CAM may not be cheap and easy to access in Europe, compared with CM as users have to personally pay for CAM and it can be difficult to access [19]. Moreover, European populations are more likely to report conventional health professionals’ recommendations for CAM use as their reason for choosing that option, compared with other global regions. This option may be because health care is readily available in most European countries; so when they have a health problem, they visit their general practitioner.

There are limited publications reporting reasons for non-use of CAM in each region. Further studies relating to this issue are required, particularly in populations from Africa, the Middle East and South America. The present systematic review found that Asian populations are more likely to question the efficacy and safety of CAM, and to be concerned about potentially harmful interactions between herbal medicines and CM. These findings imply that Asian populations seem to understand the limitations of CAM, the efficacy and safety of CAM, and are aware of herb-drug interactions.

The findings confirm that Western populations do not use CAM if they are satisfied with the efficacy and safety of CM. This outcome may be because they can easily access CM, and CAM is less likely to be considered as an option for chronic illnesses in Western countries. However, if they were to become disappointed with the CM/staff, they may decide to use CAM. This possibility is consistent with the systematic review of patients with cancer, which reported that the patients who were satisfied with CM did not use CAM [3]. Lack of reliable information about the efficacy of CAM, as a barrier to CAM use reported in the present systematic review, is consistent with the findings from a previous systematic review [9].

### Limitations of this review

Although this review only selected a small number of key words, and only three search engines in order to search the literature the findings returned 43% duplicate publications. Further reviews should search using a wide range of CAM types as keywords, e.g. yoga, acupuncture, relaxation, etc., in order to confirm the findings from the present review. There was a small number of publications addressing the reasons for CAM use in South America (n = 8); thus the findings from that continent should be interpreted with caution. This review included only publications in English; as a result the findings did not represent publications in other languages. Regarding the results from the search strategy used in this review, only 5% of the papers were excluded due to being non-English. This outcome is unlikely to have any significant impact on the findings of the present review, as most of the studies were conducted in Europe, from where a high number of publications in English were identified for inclusion in this review.

Results of publications in condition-specific populations representing a national population should also be interpreted with caution due to only 36% of these studies being designed to represent the patient population. The present systematic review found poor external validity of the included studies amongst condition-specific populations, therefore future studies should be aware of this issue.

### Impact of the findings for conventional health professionals

Expected benefits of CAM are the main reason for CAM use despite a lack of clinical trials. To promote the rational use of CAM, health care providers should be ready to provide such information to their patients and conventional medicine guidelines should report reliable information about CAM, and be easily available to, health care providers. The findings in the present review have confirmed that being disappointed with CM or associated professional providers, particularly in Western populations, is more likely to influence condition-specific populations to use CAM. To prevent patients from using CAM inappropriately, health care providers should spend more time clearly explaining treatment options, the likely treatment outcomes and potential negative effects of CAM, including herb-drug interactions.

Having an internal health locus of control seems to be a main reason for CAM use in Western populations. This finding implies that patients prefer deciding a therapy by and for themselves. To decrease inappropriate use of CAM, conventional health care providers should offer sufficient health information to their patients, as well as holding a discussion with a patient, before deciding upon a health therapy.

A person’s social network is more likely to influence their decision making regarding CAM in Asian populations. Therefore, health care providers should educate not only patients about how to properly use CAM, but also their friends and family members.

## Conclusions

It is clear that the main reasons for CAM use in all populations are a positive attitude toward CAM, that is the perceived benefit and safety of CAM, and a negative attitude toward CM, a dissatisfaction with CM. Having an internal health locus of control is a more frequently reported reason for CAM use in Western populations, whilst being influenced by social networks is a common reason for its adoption amongst Asian populations. Affordability, easy access to CAM and tradition are the most common reasons amongst African populations. Negative attitudes towards CAM and satisfaction with CM are more likely to be the reason for non-use. Conventional health professionals should acknowledge that people may turn to CAM in order to serve their needs. Therefore, health care providers should regularly ask their patients about their use of CAM before that providers prescribes any conventional medicines, in order to prevent undesirable adverse effects or CAM-drug interactions. Further studies are required to investigate reasons for CAM use in South America and reasons for non-use in all global regions, in order to provide more conclusive evidence in this field.

## Supplementary Information

**Additional file 1.** Risk of bias.

## Data Availability

The included publications in this systematic review were assessed their risk of bias in order to evaluate the quality of publications. This information provided in its supplementary information file. The other datasets used and analysed during the current study are available from the corresponding author on reasonable request.

## Abbreviations

CAM: Complementary and Alternative Medicine
CM: Conventional Medicine
HIV: Human Immunodeficiency Virus
PRISMA: The Preferred Reporting Items for Systematic Revews and Meta-Analyses
UK: The United Kingdom
US: The United States
